# Small Bowel Obstruction Secondary to Interstitial Hernia: Laparoscopic Approach

**DOI:** 10.1155/2015/780980

**Published:** 2015-10-21

**Authors:** J. M. Alvarez Gallesio, F. Schlottmann, E. E. Sadava

**Affiliations:** Division of Abdominal Wall Surgery, Department of General Surgery, Hospital Alemán of Buenos Aires, Avenida Pueyrredon 1640, C1118AAT Buenos Aires, Argentina

## Abstract

Interstitial hernias are a rare entity. Most of them are detected incidentally on imaging studies. We present a case of abdominal bowel obstruction secondary to interstitial hernia on the fifth postoperative day of an open incisional hernia repair. Laparoscopy confirmed the diagnosis and led to an accurate treatment avoiding a new laparotomy. In this case, prompt surgical decision based on clinical and CT scan findings allowed a mini-invasive approach with satisfactory outcome.

## 1. Introduction

Interstitial hernia is one in which the hernia contents are located between the layers of the abdominal wall and is generally associated with congenital defects. It was first described by Bartholin in 1661 as a variation of inguinal hernia [1]. This type of hernia is rare, and it can be easily overlooked in the setting of a patient with abdominal pain and suspicion of small bowel obstruction. We report a case of bowel obstruction secondary to postoperative interstitial hernia treated by laparoscopic approach.

## 2. Case Report

**Figure 1:**
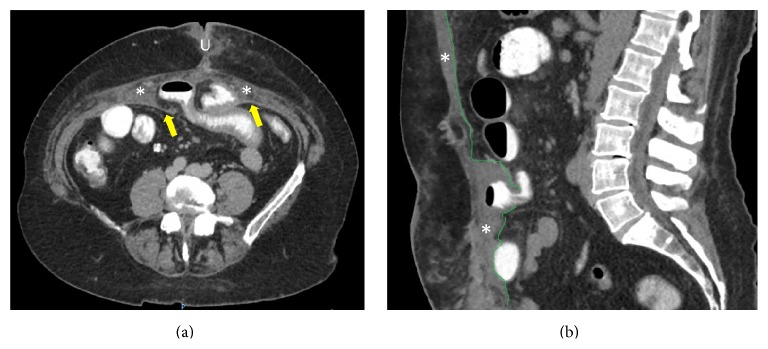
CT scan with intravenous and oral contrast. (a) Axial view. Defect in the posterior rectus sheath (arrows) with small bowel loops between parietal layers. (*∗*) Rectus abdominis muscle. U: umbilicus. (b) Sagittal view shows distension of proximal bowel loops consistent with a small bowel obstruction.

**Figure 2:**
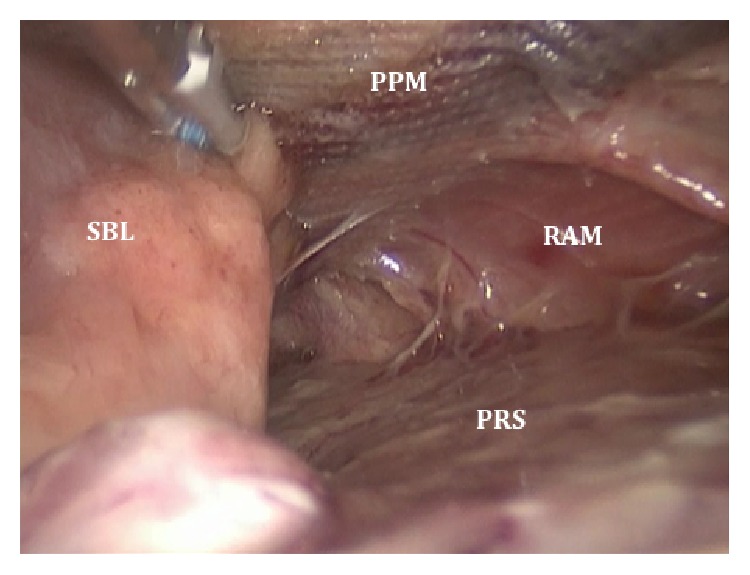
Laparoscopic view of the interstitial hernia. Small bowel loop (SBL) inside the interstitial space between polypropylene mesh (PPM) and rectus abdominis muscle (RAM) above and posterior rectus sheath (PRS) below.

**Figure 3:**
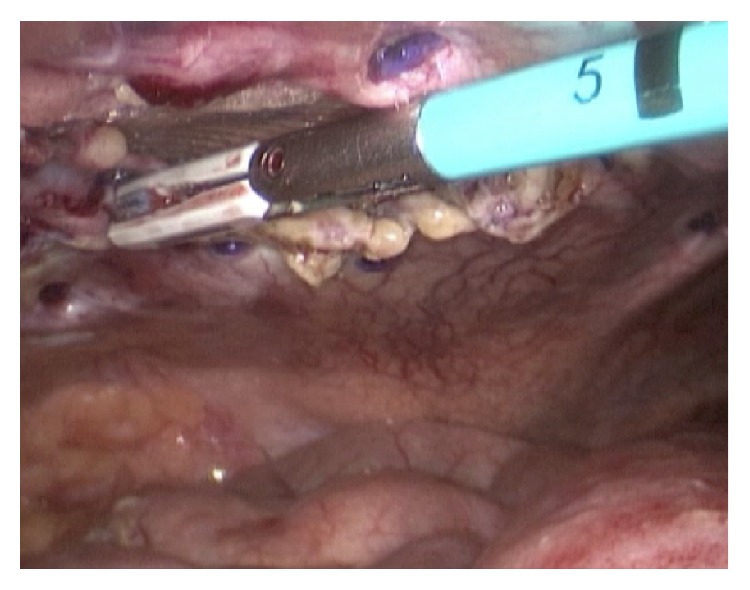
Uncovered mesh after posterior sheath fixation with absorbable tackers. The defects were measured.

A 49-year-old woman was admitted for a large ventral incisional hernia repair. Medical records showed obesity (BMI: 32 kg/m^2^), orthotopic liver transplantation for alcoholic cirrhosis (2013) and hysterectomy with bilateral salpingo-oophorectomy for endometrial cancer (2014), and recent consultation for pain secondary to noncomplicated incisional hernia but altering her quality of life. Preoperative assessment for cardiac and pulmonary function was suitable. An open ventral incisional hernia repair was performed through a xyphopubic incision and a polypropylene mesh was used in a sublay fashion. The polypropylene mesh was placed in the plane between posterior rectus muscle sheath and rectus abdominis muscle, as in Rives-Stoppa procedure. She was discharged at the first postoperative day. On the fifth postoperative day she was readmitted for increasing abdominal pain with nausea and vomiting. Computed tomography (CT) scan revealed a defect in the posterior layer of the rectus abdominis muscle sheath, with jejunal loops passing through a gap between the posterior sheath and the mesh, which was fixed to the rectus abdominis muscle (Figure 1). Surgical treatment was decided and laparoscopic approach was performed by the same surgical team. The small bowel loops were visualized between abdominal wall layers (Figure 2). Careful soft traction maneuvers were initiated and executed and after the complete reduction of unharmed intestine we observed two defects in the posterior layer of the rectus sheath, with a diameter of 3 and 4 cm. Interstitial space (between polypropylene mesh and posterior sheath of rectus abdominis muscle) was explored verifying correct mesh fixation. The detached posterior sheath was fixated to the abdominal wall with absorbable tackers, reinforcing the rim of the defects with double crown technique (Figure 3). Finally, the greater omentum was relocated to cover the exposed mesh in order to reduce visceral adhesions. The postoperative course was uneventful and patient was discharged at the second postoperative day. At the six-month follow-up there were no signs of recurrence.

## 3. Discussion

Incarcerated hernias are not uncommon; an incidence up to 19% in emergent ventral hernia repair has been published in the last decade [2]. In addition, these cases are challenging given the fact that they have an increased morbidity and mortality compared to noncomplicated hernias. Laparoscopic approach has gained popularity in small bowel obstruction management in selected patients [3, 4]. It offers a better view of the abdominal cavity compared to the open approach and the possibility to achieve an adequate treatment in a less invasive way in most cases. On the other hand, a delay in decision-making could disfavour this approach given that markedly distended bowel loops preclude accurate diagnosis and treatment [5].

Given the low frequency of interstitial hernias, most of them are incidentally diagnosed on imaging studies or during surgical exploration. However, CT scan is considered the best diagnostic study for this rare entity [6]. In this case, CT scan findings led us to suspect an interstitial hernia and laparoscopy confirmed the diagnosis.

Another rare entity known as posterior rectus sheath hernia has been reported in the literature, although it is not clearly defined. This type of hernia has been described a few times since the first report by Lopez in 1937 [7, 8]. It is more frequent in women with a mean of 50 years. Most of them are either posttraumatic or postsurgical, but none has been described in the postoperative period of incisional ventral hernia repair. In our case we prefer to use the term interstitial hernia because a new virtual space was created during the open surgery (Stoppa procedure) to position the mesh, which was occupied by hernia contents probably due to acute tears in the posterior rectus sheath. Conversely, the posterior rectus sheath hernia seems to be a chronic entity.

Overall, there is scarce scientific literature regarding interstitial hernia and even less about interstitial hernia following a surgical procedure. Although the closure of the posterior sheath of the rectus abdominis muscle and peritoneum is generally tension-free, we believe that small tears on these layers may be frequent after incisional hernia repair but most of them have uneventful course. Therefore, we deduct that an increase in the abdominal wall tension during the postoperative period could tear these thin layers and generate an interstitial space leading to small bowel obstruction.

Patient comorbidities could be a potential cause of increased intra-abdominal pressure and weakening of the tissues. It is well known that obesity is a major risk factor of recurrence and complications after incisional hernia [9, 10]. A recent study described incisional hernia volume/peritoneal volume ratio (Tanaka Index) <20% as predictive factor for tension-free fascia closure [11]. In our case a ratio of 9% was observed on the preoperative CT scan. In addition, our patient was treated with immunosuppressive drugs (rapamycin and corticosteroids) because of the liver transplantation before the incisional hernia repair surgery. Some authors describe that rapamycin impairs fibroblasts and inhibits angiogenesis [12, 13]. Therefore, a negative impact on wound healing should also be expected.

**Figure 4:**
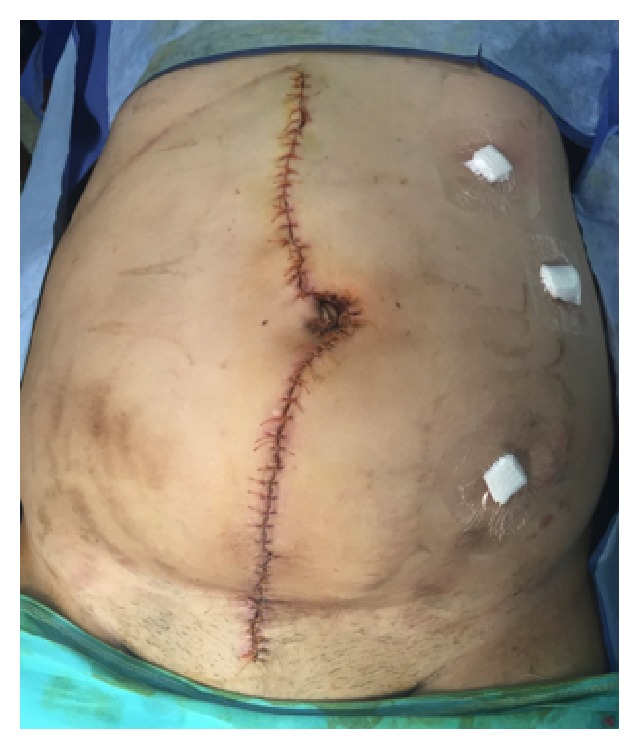
Postoperative view after laparoscopic surgery.

Lastly, the impact of hernia recurrence after laparoscopic approach in patients with previous open incisional repair is unknown. However, laparoscopy would lead us to an accurate diagnosis of acute interstitial hernia. During laparoscopy we observed two defects in the posterior layer of the rectal abdominis muscle sheath, with bowel loops trapped between abdominal wall layers. This approach averted a new xyphopubic laparotomy and the need of mesh detachment or even replacement (Figure 4).

## 4. Conclusion

Interstitial hernia is a rare entity and is even more unusual after an incisional hernia repair. It can be easily misdiagnosed and CT scan is the best diagnostic study. This entity should be considered in patients who undergo abdominal wall surgery and develop bowel obstruction during the postoperative period. Laparoscopic approach allows an accurate diagnosis and treatment avoiding new laparotomies and their potential complications.
